# Alcohol Dependence Among Adult Males in Chengalpattu District, South India: A Mixed Methods Study

**DOI:** 10.7759/cureus.66624

**Published:** 2024-08-11

**Authors:** Gayathri Baskaran, Anantha Eashwar V. M., Stephen T., Meena Priya S., Hariharan Surathkumaar, Charu Latha

**Affiliations:** 1 Community Medicine, Sree Balaji Medical College and Hospital, Chennai, IND; 2 Preventive Medicine, Dr. MGR Educational and Research University, Chennai, IND

**Keywords:** substance recreational use, insomnia disorder, job stress, peer pressure, suicide and depression, alcohol dependence

## Abstract

Introduction

Post-pandemic alcohol consumption is on the rise due to people starting to adapt themselves to the practice of consuming alcoholic beverages at home. In addition to the direct effects of intoxication and addiction, estimates suggest that alcohol contributes to approximately 20-30% of global cases of oesophagal cancer, liver cancer, cirrhosis of the liver, homicide, epilepsy, and motor vehicle accidents. In India, one-fifth of alcohol consumers were found to be alcohol dependent. The study was done with the primary objective of finding out the prevalence of alcohol dependence among alcohol users and exploring the reasons for alcohol dependence among alcohol users in an urban area of Chengalpattu District, Tamil Nadu.

Methodology

The study design is an explanatory sequential mixed-methods study. It was done among 624 adult male alcohol consumers in the Chennai district, selected by the cluster sampling method in a community setting. The Alcohol Use Disorders Identification Test (AUDIT) was used to diagnose alcohol dependence. Using the purposive sampling method, in-depth interviews were conducted among 24 alcohol-dependent people to explore and understand their experiences, identify common themes, and provide insights into the problem. Quantitative data were analysed using Statistical Package for Social Sciences (SPSS) version 26 (IBM Corp., Armonk, NY), and qualitative data were analysed using deductive content analysis using Qualcoder software.

Results

The mean age of the study participants was 38±7 years. Among current alcohol consumers, 16.9% (106/624) were found to be suffering from alcohol dependence. The significant predictors of alcohol dependence were found to be unskilled occupation (adjusted odds ratio [AOR] = 2.09), having suicidal ideation (AOR = 2.4), alcohol consumption by family members (AOR = 1.90), depression (AOR = 3.98), drinking pattern-affected interpersonal relationships (AOR = 2.29), and not receiving health education about alcohol use in school/college (AOR = 1.74). The major themes and codes identified among alcohol dependents were factors related to mental health, physical health, and social factors.

Conclusion

This study provides essential points of reference for policymakers and primary care physicians to develop prevention strategies for people to understand and overcome the problem of alcohol addiction, and it also sheds light on the burden of alcohol dependence and their lived experiences.

## Introduction

Alcoholic beverages contain ethanol, which is a psychoactive and toxic substance with dependence-producing properties. Alcohol has been widely used in many cultures for centuries, but it is associated with significant health risks and harm [1]. Traditional alcoholic beverages contain about 20% to 40% alcohol, whereas it is as high as 56% in illicit liquor, thus making the latter a big menace to the country’s health. Adding to the fire, illicit liquor is relatively cheaper than licenced country liquor and, therefore, more rampant among urban and rural poor communities, making them more vulnerable. In India, around one-fifth of alcohol consumers were found to be suffering from alcohol dependence [2,3].

The World Health Organization’s (WHO) International Classification of Diseases (ICD-11) and the American Psychiatric Association Diagnostic and Statistical Manual of Mental Disorders, Fifth Edition (DSM-5) set out guidelines for identifying alcohol dependence or moderate to severe Alcohol Use Disorders (AUDs) [4]. DSM-5 integrates the two Diagnostic and Statistical Manual of Mental Disorders, Fourth Edition (DSM-IV) disorders, alcohol abuse, and alcohol dependence, into a single disorder called AUD with mild, moderate, and severe sub-classifications. The diagnosis of AUD is established using the criteria in the DSM-V [5]. These criteria, gleaned from clinical history and collateral sources, generally assess the impact of alcohol on a patient’s relationships, health, activities, and ability to moderate their drinking [6].

Edwards has described an 'alcohol dependence syndrome (ADS)', which consists of several elements that characterize the clinical picture of alcohol dependence [7]. It involves a loss of control over the ability to drink moderately. This loss of control results in negative consequences that impact relationships, physical and mental health, and the ability to fulfil roles and obligations. Alcohol is used in increasing amounts to achieve the same effect, a phenomenon known as tolerance, and its absence results in withdrawal symptoms. Patients also experience intense cravings for alcohol that drive ongoing consumption [8].

Alcohol causes the release of dopamine in the ventral tegmental area, which is a part of the reward pathway. Alcohol also affects other reward systems, such as the endogenous opioid system, the γ-aminobutyric acid (GABAergic) system, glutamate, and serotonin. The reinforcing effects of alcohol include the ability to induce euphoria and anxiolysis. The higher the consumption of alcohol, the greater the prevalence of ADS and more stress to the individual, their family members, and society as well [9].

Based on the above background, the study was done with the primary objective of finding out the prevalence of alcohol dependence among alcohol users and exploring the reasons for alcohol dependence among alcohol users in an urban area of Chengalpattu District, Tamil Nadu.

## Materials and methods

Study design

The study design is an explanatory sequential mixed method.

Quantitative component

Study Design

This is a cross-sectional study.

Study Population

Adult males above 18 years of age residing in Anakaputhur, an urban area of Chengalpattu district, Tamil Nadu.

Sample Size

A previous study by Eashwar et al. found the prevalence of alcohol dependence to be 14.7%. Applying this prevalence as P in the formula 1.96 × 1.96 × *P* × *Q*/*L*2 with an absolute precision (L) of 3% gives a required sample size of 624 [10].

Sampling Method

A district-wide survey conducted by the Urban Health Training Centre field staff attached to a tertiary medical college in Chengalpattu district found that Anakaputhur district had 2543 male alcohol consumers divided among four wards. Based on the name list available, 156 adult male alcohol consumers were selected randomly from each ward by simple random sampling using a random number generator to reach the required sample size of 624.

Study Tool

Depression was assessed by administering the nine-item Hamilton Depression Scale (HAM-D) questionnaire. It is a widely used and validated tool that is used in clinical settings to diagnose depression. It has 17 items. The scoring ranges are as follows: 0-7 indicates no depressive symptoms; 8-16 signifies mild depression; 17-23 represents moderate depression; and scores over 24 denote severe depression. A score above 8 was taken as a cut-off for depression in the present study [11]. The Alcohol Use Disorders Identification Test (AUDIT) was used to diagnose alcohol dependence. It is a 10-item questionnaire developed by the WHO that is used as a screening tool among alcohol consumers to diagnose alcohol-related problems. The minimum score that could be obtained was 0, and the maximum score was 40. A cut-off score above 20 was taken as a diagnostic of alcohol dependence [12].

Inclusion and Exclusion Criteria

Adult males above 18 years of age who consumed alcohol in the past six months and are current consumers and persons with co-existing psychiatric illnesses like schizophrenia and bipolar disorder were excluded from the study.

Data Analysis

Data were entered in Microsoft Excel (Microsoft® Corp., Redmond, WA) and analysed using SPSS software version 26. Descriptive statistics were presented as frequency and percentage. The association between alcohol dependence and related variables was calculated using chi-square. The Enter method of binomial logistic regression analysis used variables that were statistically significant in bivariate analysis.

Qualitative component

Study Population

Adult males aged above 18 years of age and having alcohol dependence, according to the AUDIT questionnaire, who live in the urban area of Chengalpattu district, Tamil Nadu, were included.

Sampling Type

A purposive sampling method was used to select the alcohol consumers based on their AUDIT score (score above 20).

Study Tool

The study tool is an unstructured interview schedule that utilizes guided questions.

Data Collection and Analysis

In-depth interviews were recorded, transcribed verbatim, and uploaded into the Qualcoder software. Deductive content analysis was used to perform thematic coding to identify themes, subthemes, and codes. Data saturation was reached after 24 in-depth interviews.

Ethical Approval and Informed Consent

Ethical approval was obtained from the Institutional Human Ethical Committee of a tertiary medical college in Chengalpattu district (approval number: SBMCH/IHEC/2023/96, approval date: June 11, 2023). Informed consent was obtained from each study participant before enrolment.

## Results

Table 1 shows the socio-demographic characteristics of the study population. Around 75% (464/624) of the study population was under 45 years of age. Almost 28.8% (180/624) of the population were graduates, and 68.6% (428/624) were married. Regarding socioeconomic status, 30.8% (192/624) belonged to the middle socio-economic class, according to the modified BG Prasad classification.

**Table 1:** Socio-demographic details of study participants

S. No	Sociodemographic details	Frequency (N = 624) (n)	Percentage (%)
1.	Age		
	<30	216	34.6
	31–45	248	39.7
	46–60	132	21.2
	>60	28	4.5
2.	Education		
	Illiterate	108	17.3
	Primary school	112	17.9
	Secondary school	60	9.6
	Higher secondary	164	26.3
	Graduate or above	180	28.8
3.	Occupation		
	Unskilled worker	156	25
	Skilled worker	256	41
	Professional	96	15.4
	Semi-professional	84	13.5
	Clerk	12	1.9
	Unemployed	20	3.2
4.	Marital status		
	Married	428	68.6
	Unmarried	128	20.5
	Widower	60	9.6
	Divorced	8	1.3
5	Socio economic status		
	Upper class	148	23.7
	Upper middle class	156	25
	Middle class	192	30.8
	Lower middle class	96	15.4
	Lower	32	5.1

Table 2 shows the association between alcohol dependence and related variables. On binomial logistic regression analysis, variables that were found to have a statistically significant association with alcohol dependence were unskilled occupation (AOR: 2.09, 95% CI: 1.23-3.56), having suicidal ideation (AOR: 2.40, 95% CI: 1.38-4.16), alcohol consumption by family members (AOR: 1.90, 95% CI: 1.07-3.39), personal relationships getting affected due to alcohol use (AOR: 2.29, 95% CI: 1.28-4.08), not receiving health education about alcohol use in school or college (AOR: 1.74, 95% CI: 1.01-3.00), and possible depression according to the HAM-D scale (AOR: 3.98, 95% CI: 2.46-6.43). In the Hosmer-Lemeshow goodness-of-fit test, the P-value obtained was above 0.05, which indicated that the model fit the data well.

**Table 2:** Logistic regression analysis between alcohol dependence and related variables *P<0.05, statistically significant at a 95% confidence interval. CI: confidence interval. Unadjusted odd's ratio was calculated using chi-square. Adjusted odd's ratio was calculated using the enter method of logistic regression.

S. No	Variable	Alcohol dependence		Total N = 624 n (%)	Unadjusted odd’s ratio (95% CI)	Adjusted odd’s ratio (95% CI)	P-value
		Yes, n = 106, n (%)	No, n = 518, n (%)				
1	Marital status						
	Unmarried	83 (19.4)	345 (80.6)	428 (68.6)	1.80 (1.10–2.97)	0.61 (0.34–1.07)	0.088
	Married	23 (11.7)	173 (88.3)	196 (31.4)	Reference category		
2	Occupation						
	Unskilled Worker	46 (26.1)	130 (73.9)	176 (28.2)	2.28 (1.48–3.52)	2.09 (1.23–3.56)	0.006*
	Skilled Worker	60 (13.4)	388 (86.6)	448 (71.8)	Reference category		
3	Suicidal Ideation						
	Yes	36 (31.0)	80 (69)	116 (18.6)	2.816 (1.76–4.49)	2.40 (1.38–4.16)	0.002*
	No	70 (13.8)	438 (86.2)	508 (81.4)	Reference category		
4	Alcohol consumption by family members						
	Yes	79 (23.0)	265 (77.0)	344 (55.1)	2.79 (1.74–4.46)	1.90 (1.07–3.39)	0.028*
	No	27 (9.6)	253 (90.4)	280 (44.9)	Reference category		
5	Domestic violence following alcohol use						
	Yes	53 (22.5)	183 (77.5)	236 (37.8)	1.83 (1.20–2.78)	1.26 (0.74–2.13)	0.38
	No	53 (13.7)	335 (86.3)	388 (62.2)	Reference category		
6	Personal relationships are affected by alcohol use						
	Yes	84 (22.6)	288 (77.4)	372 (59.6)	3.049 (1.84–5.02)	2.29 (1.28–4.08)	0.005*
	No	22 (8.7)	230 (91.3)	252 (40.4)	Reference category		
7	Not received health education about alcohol use in school/college						
	No	72 (20.5)	280 (79.5)	352 (56.4)	1.80 (1.15–2.80)	1.74 (1.01–3.00)	0.044*
	Yes	34 (12.5)	238 (87.5)	272 (43.6)	Reference category		
8	Depression (HAM-D)						
	Yes	66 (35.9)	118 (64.1)	184 (29.5)	5.59 (3.59–8.71)	3.98 (2.46–6.43)	0.02*
	No	40 (9.1)	400 (90.9)	440 (70.5)	Reference category		
9	Duration of the current pattern of drinking						
	>15 years	37 (22)	131 (78)	168 (26.9)	1.58 (1.01–2.47)	1.68 (0.99–2.85)	0.054
	<15 years	69 (15.1)	387 (84.9)	456 (73.1)	Reference category		

Table 3 shows the themes, subthemes, and codes that were obtained from alcohol-dependent persons using qualitative interviews. The major themes that were identified were related to mental health, physical health, and social factors, which the participants perceived as the causes of dependent alcohol drinking patterns.

**Table 3:** Themes, sub-themes, and codes obtained from alcohol-dependent persons (N = 24)

S. No	Themes	Sub-themes	Codes	Quotes
1	Mental health	Suicidal ideation	Thoughts about ending your own life if you have not consumed alcoholic beverages	“If not for the alcoholic beverages, I would have been dead a long time ago. It is what keeps my sanity in check. After a long, hectic day at work, I drink alcoholic beverages to unwind myself and cool my nerves. It reduces all of my worries and stress.”
		Refusing to accept that they have a drinking problem	Normalizing the drinking pattern as normal	
		Stress	Feels that alcohol consumption will make the person feel calm and relaxed	
		Sleep problems	It feels that alcohol consumption will help improve sleep and help overcome sleep problems	
2	Physical health	Lack of awareness	Unaware of the problems that alcohol use could cause to the individual	“Drinking was the only way for me to cope with the chronic back pain I developed three years ago. It was highly effective at first. But eventually, it made my pain worse and led to many health problems.”
		Body pain	Feels that alcohol consumption will numb the body’s pain and make the person feel better	
3	Social Factors	Peer pressure	He feels that he needs to consume alcohol to survive in the workplace environment	“Alcohol is one of the major means I enhance my social relationships. It helps me feel connected with my teammates, which I feel is essential to survive in my workplace.”
		Recreational	Mandatory alcohol parties in the workplace weekly or monthly	
		Acceptance in family	Wife/parent’s acceptance of alcohol use	

Mental health

Suicidal Ideation

Most of the participants felt that they would end up with suicidal thoughts if they stopped consuming alcoholic beverages. One 35-year-old participant said, “If not for the alcoholic beverages, I would have been dead a long time ago. It is what keeps my sanity in check.”

Refusing to Accept That They Have a Drinking Problem

All the study participants refused to accept that they had a drinking problem. A 41-year-old participant said, “In this current generation, drinking is part of our lifestyle. I see no harm in using alcohol to relax and celebrate my life.”

Stress

Around half of the study participants reported that they drink alcohol to cope with their stress. One of the participants said, “After a long, hectic day at work, I drink alcoholic beverages to unwind myself and cool my nerves. It reduces all of my worries and stress.”

Sleep

The majority of the study participants consumed alcoholic beverages daily at night to fall asleep. They also subjectively felt that it affected their quality of sleep. A 28-year-old participant said, “Initially, consuming alcoholic drinks to sleep worked wonders, and I was able to get quality sleep. But as months passed by, the quantity of alcohol required to sleep increased to attain the necessary effect, which in turn affected my quality of sleep.”

Physical health

Lack of Awareness

Around one-third of the study participants were not fully aware of the consequences of alcohol consumption. A 45-year-old participant reported, “I thought a drink or two every day would not cause me any harm. During a routine health checkup, when the doctor pointed out that most of my health issues were due to my drinking pattern, I learnt about the long-term effects of alcohol consumption.”

Body Pain

Almost all of the study participants who were working in an unskilled cadre of work responded that alcoholic beverages helped them numb the physical pain endured in their workplace (body pain and joint pain). “Drinking was the only way for me to cope with the chronic back pain I developed three years ago. It was highly effective at first. But eventually, it made my pain worse and led to many health problems.”

Social factors

Peer Pressure

The compulsion to consume alcoholic beverages at social gatherings, parties, etc. was one of the many vital reasons quoted by the participants that led to the development of dependent drinking patterns. One of the participants, who had consumed alcohol since he was 17 years old, reported, “I didn’t even like the smell or taste of alcohol. But my friends compelled me and encouraged me to drink. Since I did not want to be left out of my friend’s circle, I started drinking, which I am unable to stop now.”

Recreational Purposes

Participants reported that workplace alcohol parties were the standard norm, which happened at least twice a week. One of the participants stated, “Alcohol is one of the major means I enhance my social relationships. It helps me feel connected with my teammates, which I feel is essential to survive in my workplace.”

Acceptance in the Family

Participants felt that their wives and family members expressed little opposition to their drinking patterns, as they accepted their drinking behaviour if it did not cause them harm. A 27-year-old participant stated, “My wife pours me drinks in the night after my children sleep. She makes me a suitable dinner, which goes along with my drinks. I believe this is why I feel my drinking habits are accepted and won't lead to any harm.”

## Discussion

The consumption of alcohol has claimed the lives of many individuals and broken families. Even though the harmful effects of alcohol consumption are well documented and studies are well known, alcohol consumption is still rampant in our country. The study was done to find out the prevalence of alcohol dependence among alcohol consumers and to explore the reasons why people end up as dependent alcoholics, which are discussed below compared with the results of studies done elsewhere.

The present study found the prevalence of alcohol dependence among alcohol consumers to be 16.9%. A study done by Avasthi et al. in Punjab found the prevalence of alcohol dependence to be 10.9% [13]. In a study done by Rathod et al. in Madhya Pradesh, the prevalence was 5.5% [14]. In Tamil Nadu, a study by Eashwar et al. found the prevalence to be 14.7% [10]. Though alcohol consumption in India is low when compared to developed countries like America and the United Kingdom, the proportion of dependent alcoholics is higher, with one-fifth of alcohol consumers in India being dependent alcoholics [15]. This is attributed to various factors like socio-economic status, the easy availability of alcoholic beverages, and the lack of proper regulations governing alcohol use in certain states of India [16].

There was a statistically significant association between unskilled workers and alcohol dependence. Similar results were obtained in a study done by Ezhumalai et al., in which alcohol dependence was more common among people involved in unskilled labour and abstinence was associated with skilled labour [17]. This shows that suitable job placements and frequent alcohol health education activities targeted towards workers involved in unskilled labour could bring down the problem of alcohol dependence.

Among the alcohol consumers who had suicidal ideation, around 31% were alcohol dependents, and the association was also found to be statistically significant. In a study done by Rahoof et al. and Agarwal et al., suicide was found to be high among alcohol consumers who were dependent alcoholics [18,19]. This warrants the need for suicide risk assessment and prevention strategies to be the main strategy when handling alcohol-dependent people in primary care settings.

The present study found a statistically significant association between alcohol dependence and alcohol consumption among family members. Similarly, those who had their relationships affected by alcohol use were also associated with alcohol dependence. Similar results were obtained in a study done by Chinnusamy et al., in which alcohol use among family members played a significant role in alcohol dependence. Also, it was found that alcohol dependence leads to increased interpersonal conflict with family members, financial problems, and domestic violence [20].

In the present study, those with alcohol dependence were at increased odds of having comorbid depression. Several literature have pointed out the link between depression and alcohol dependence [21,22]. Depression in a dependent alcoholic will not only lower his willpower to resist alcohol use, but he may also use it to relieve his depressive symptoms, as evidenced by the qualitative finding in this study, in which alcohol was used as a significant means to relieve stress. Hasin and Grant [23] observed similar findings.

One of the significant subthemes identified under the mental health theme was that alcohol-dependent people refused to recognize that they had a drinking problem. Also, they were unaware of the health problems alcohol could bring about in their system. In a study done by Schuckit et al., most of the dependent alcoholics did not identify themselves as having a drinking problem, provided misleading answers and defended their drinking patterns [24]. This denial pattern warrants the use of behaviour change communication and health education activities targeted towards alcohol consumers so that they will understand and come out with their alcohol drinking problem.

During in-depth interviews, many participants cited the use of alcohol to fall asleep at night. They also accepted that, though it helps them to fall asleep, consuming alcohol significantly reduces their quality of sleep. Similar results were obtained in a study by Brower [25]. Using alcohol for recreational purposes and due to peer pressure was one of the major themes identified by the interviews. Studies done by Studer et al. and Patrick et al. found that peer influence and social drinking are two of the significant reasons for alcohol misuse, leading to alcohol addiction [26,27].

The study's major limitation is the response bias, which would have occurred when interviewing alcohol consumers. Since it was a community-based study, participants may have underreported their pattern of alcohol consumption and provided socially desirable responses to present themselves in a favourable situation. Despite providing a non-judgemental environment, it may have been challenging to eliminate bias.

## Conclusions

The findings of the present study highlight the need for holistic, multifaceted approaches to address the problems of alcohol dependence. Interventions should consider the psychological and social dimensions of alcohol dependence. Tailored programs that provide emotional support, enhance coping strategies, and facilitate social reintegration are crucial for effective treatment and sustained recovery. This study underscores the complexity of alcohol dependence and the necessity for comprehensive, empathetic, and individualized approaches in both prevention and treatment.
